# Scoliotic posture as the initial symptom in adolescents with lumbar disc herniation: its curve pattern and natural history after lumbar discectomy

**DOI:** 10.1186/1471-2474-12-216

**Published:** 2011-09-30

**Authors:** Zezhang Zhu, Qinghua Zhao, Bin Wang, Yang Yu, Bangping Qian, Yitao Ding, Yong Qiu

**Affiliations:** 1 Spine Surgery, Drum Tower Hospital, Nanjing University Medical School, Joint Scoliosis Research Center of Nanjing University and the Chinese University of Hong Kong. 321 Zhongshan Road, Nanjing 210008, China; 2 Department of Surgery, Drum Tower Hospital, Nanjing University Medical School. 321 Zhongshan Road, Nanjing 210008, China

**Keywords:** Scoliosis, Adolescent, Lumbar disc herniation, Discetomy

## Abstract

**Background:**

There have been few studies focusing on the curve pattern of scoliosis caused by lumbar disc herniation (LDH) in adolescents and the natural history of scoliosis after discectomy. The current study was carried out to identify the curve pattern of scoliosis and investigate the effect of posterior discectomy on the curve improvement in adolescents with LDH.

**Methods:**

This review focused on a group of 26 adolescents with LDH who initially presented to our clinic for evaluation of scoliosis, followed by posterior discectomy between 2000 and 2009. Radiographic measurements included curve pattern, specific curve features, trunk shift, and sagittal profile. The correlation between the side of disc herniation and the direction of lumbosacral curve and the trunk shift was evaluated.

**Results:**

A typical curve pattern was initially identified in all of the patients as a short lumbosacral curve accompanied with a long thoracic or thoracolumbar curve toward the opposite side. 23 of 26 patients (88.5%) had a trunk shift more than 2.0 cm away from the midline, showing a poor coronal balance. A relatively straight sagittal profile was noted in all the patients. 84.6% (22/26) patients had a disc herniation at the convex side of lumbosacral curve. Similarly, 73.1% (19/26) patients showed a trunk shift toward the opposite side of disc herniation. All of the patients had an marked curve improvement immediately after discectomy. In the 17 patients with a more than 2-year follow-up, only two had a residual lumbosacral curve greater than or equal to 20 degrees. The mean ODI improved from 21.4% before surgery to 7.3% at the final follow-up.

**Conclusions:**

A short lumbosacral curve accompanied with a long thoracic or thoracolumbar curve toward the opposite side, and a relatively straight sagittal profile have been noted in all the patients. The direction of lumbosacral curve and trunk shift was related to the side of disc herniation. A majority of patients have a small curve size while assosiated with a significant coronal imbalance. Earlier decompression can provide a greater opportunity for spontaneous correction of scoliosis.

## Background

Scoliotic posture has been found in coincidence with lumbar disc herniation (LDH) in both adolescents and adults [1-3]. Although the pathophysiology is not fully understood, scoliotic posture has been considered by most authors as a compensatory attempt of the body to relieve nerve irritation. Previous studies have reported that scoliotic posture would be improved once the painful stimulus is removed [1,2]. LDH is very unusual in adolescents based on population-based studies. Since Wahren firstly reported a case of a disc herniation surgery in a 12 year-old boy, some single-case or series reports revealed the incidence varying from 1% to 5% in adolescent population [4,5]. Although genetic predisposition [6,7] and trauma [8,9] have been reported as causes of LDH in adolescents, the etiology remains an area of continuing debate. The clinical features of LDH in adolescents, characterized with broader spectrum of symptoms, are typically different from those in adults [10,11]. Besides low back pain and leg pain, scoliotic posture, difficulty in walking and paravertebral muscle spasm are also frequent complaints in children and adolescents with LDH. Even in some patients, the scoliotic posture is their first symptom for clinical consultation, which can be attributed to the anatomical feature that pediatric spines have better adaptive capacity to protect nerve tissue via lateral flexion [3]. To our knowledge, there have been few studies focusing on the curve pattern of scoliotic posture caused by LDH in a young population, especially in those with an obvious spinal deformity as their chief complaint. The present study retrospectively reviewed an important group of adolescents with LDH who initially presented scoliotic posture for clinical evaluation, aiming to elaborate their curve features of scoliosis and investigate the effect of posterior discectomy on the curve improvement.

## Methods

This review focused on a group of 26 adolescents with LDH who initially presented to our clinic for evaluation of scoliotic posture between January 2000 and May 2009. There were 18 males and 8 females with a mean age of 17.7 years (range, 14.3-20.0 years). Three patients had a Risser grade of 3 while the others showed a Risser sign 4 or more. Nine patients had a history of significant trauma. Four patients had been misdiagnosed as idiopathic scoliosis managed with bracing for 6-15 months at local hospitals. There was no definite family history of scoliosis in this series. The duration between the onset of scoliotic posture and presentation at our clinic ranged from 10 days to 15 months (mean, 4.3 months). Based on detailed history-taking and physical examination, some other symptoms and signs were revealed (Table 1). Except for 16 patients having limitation of lumbar motion, an Adams forward bend test was performed on the other 7 patients to differentiate scoliotic posture from structural scoliosis. The back deformity disappeared during the test in all of them, showing a nonstructural pattern. Lumbar disc herniation was confirmed by means of computed tomography (CT) or magnetic resonance imaging (MRI). The disc herniation located at L4-L5 in 14 patients, at L5-S1 in 6, and at 2 levels of L4-L5 and L5-S1 in 6. Totally, herniated disc was found in 32 levels, including protruded disc in 24 levels, extruded disc in 7 and sequestered disc in 1. Herniated disc was centrolateral in 21 levels and foraminal in 11 levels. As for the 6 patients with L4-L5 and L5-S1 herniation, the two-level herniated discs located on the same side were noticed in each patient.

**Table 1:** Symptoms and signs at presentation

Clinical finding	Patients (%)
Restriction of run and jump	21 (81)
Limitation of lumbar spine movement	19 (73)
Low back pain	15 (58)
Unilateral pain or discomfort in the buttock, or thigh	9 (35)
Unilateral radiating sciatic pain	4 (15)
Scoliotic posture	26 (100)
Disappearing of scoliosis in prone position	0 (0)
Motor weakness	4 (15)
Sensory deficit	14 (54)
Positive leg-raising test	18 (69)

Radiographic measurements were made on standing anteroposterior and lateral radiographs of the entire spine before surgery, at the immediate postoperative period (just before discharge), and at the latest follow-up. Both the overall curve pattern and specific curve features (levels, magnitude, rotation, and direction) were evaluated on the anteroposterior radiographs. The magnitude of curvature was measured by Cobb method. As for a lumbosacral curve that has its apex at L5 or below, S1 was selected as the lower end vertebra for Cobb measurement. Apical vertebral rotation (AVR) was assessed using the Nash-Moe grading. On condition that the curve was too irregular to identify the exact apical vertebra, the vertebra in the apical area showing maximal rotation would be chosen for AVR measurement. Trunk shift was determined by measuring the horizontal distance between C7 plumb line and the central sacral vertical line (CSVL). By convention, shift of the C7 plumb line to the left is considered negative balance, while a shift to the right is considered positive. Poor coronal balance or decompensation is defined as the value of trunk shift exceeds 2.0 cm [12,13]. On standing lateral radiographs, thoracic kyphosis and lumbar lordosis were measured using the Cobb angle. The superior end plate of T3 and inferior end plate of T12 were used to measure thoracic kyphosis [14,15]. The superior end plates of T12 and S1 were used to measure lumbar lordosis [14,15]. Values were interpreted with respect to normative data for sagittal alignment in children and adolescents. Thoracic alignment was defined as normal (20°-50°), hyperkyphotic (> 50°), and hypokyphotic (< 20°). Normal lumbar lordosis was 64° ± 10°, and accordingly lumbar alignment was defined as normal (54°-74°), hyperlordotic (> 74°), and hypolordotic (< 54°) [15].

All patients received conservative treatments consisting of analgesics, injections, bed rest, and appropriate physical therapy prior to surgery. A standard posterior micro-discectomy was performed on 21 patients following failure to respond to conservative management for 7-12 weeks. The other 5 patients (No. 2, 6, 16, 23 and 25) had pain relief after conservative treatment for 10-12 weeks, however, the surgical intervention was also indicated due to the persistence of their scoliotic posture. After surgery, the patients were kept at rest and then gradually mobilized and straight-leg raising until they were ambulatory (1-2 days after operation). On account of the limited domestic rehabilitation condition, patients were instructed to rehabilitate in our department until they were discharged 7-10 days after surgery. During the later hospital stay, they were encouraged to walk and undergo posture training with a small lumbosacral corset. An adjuvant pelvic traction during rest time was used about two weeks for those who had severe trunk shift. The pain was assessed by calculating percentage improvement. The pain was scored from 0 (no pain) to 10 (severe pain) by numeric pain scale. After 2002, the Chinese version of the Oswestry Disability Index (ODI) [16] was available, in which Section 8 (sex life) was omitted. The total score is expressed as a percentage, wherein 0% represents no pain and disability, and 100% represents the worst pain and disability.

### Statistical analysis

SPSS 14.0 package software (SPSS Inc., Chicago, IL) was used for the statistical analysis. Difference was regarded as significant when the P value was less than 0.05. The paired sample t-test was run to compare differences of Cobb angles before surgery, immediately after surgery, and at the latest follow-up. Fisher's exact test was used to assess the significance of the relationship between the convex side of lumbar curve and the side of disc herniation, the relation between the direction of trunk shift and the side of disc herniation at presentation.

## Results

**Figure 1:**
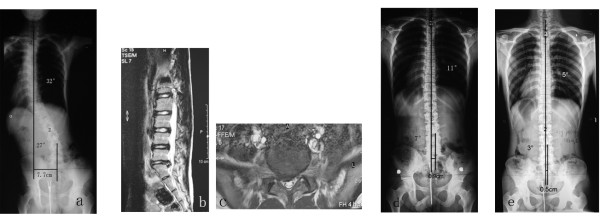
**18.4-years-old-boy with L5-S1 disc herniation and scoliosis (patient No. 16)**. (**a) **Preoperative AP radiograph showed 32° left thoracic curve (T2-L2) and 27° right lumbosacral curve (L2-S1), the trunk shift was 7.7 cm. **(b, c) **MRI revealed L5-S1 disc space narrowing and paracentral disc protrusion with impingement of the right S1 nerve root. (**d) **Immediately after surgery, radiograph showed 11° thoracic curve and 7° lumbosacral curve with the trunk shift as 0.9 cm. (**e) **Anteroposterior radiograph taken 2.5 years after surgery showed 5° thoracic curve and 3° lumbosacral curve, the trunk shift was 0.5 cm, considering as being in normal condition.

The preoperative and postoperative radiographic findings were shown in Table 2. Seventeen patients had a left-sided disc herniation, while the other 9 had a disc herniation on the right side. A common curve pattern was identified in all of the patients as following: a short lumbosacral curve accompanied with a long thoracic or thoracolumbar curve toward the opposite side (Figure 1). For lumbosacral curve, the preoperative Cobb angle was 10° to 29° (mean, 19.5°). Three to 6 levels (average, 4.5 levels) were involved with variability of the upper end vertebra (L1-L4), most commonly found at L2 (53.8% of cases), while the lower end vertebra was constant at S1. As for the proximal curve, curve magnitude ranged from 14° to 35° (mean, 24.7°). A thoracic curve was noted in 23 patients (88.5%), in comparison to a thoracolumbar curve in only 3 patients. The proximal curve involved 7 to 14 levels (mean, 10.7 levels) with variability in the proximal (T2-T8) and distal (L1-L4) end vertebrae. Most of the proximal curves (80.7%, 21/26) were irregular, characterized by difficulty in identifying the exact apex. The mean AVR was 0.54 (range, 0-1) in the proximal curves, while 0.19 (range, 0-1) in the lumbosacral curves, showing the apical vertebrae featured with very small rotation or neutral position. The trunk shift was noted from 0.9 cm to 7.7 cm (mean, 3.7 cm). Twenty-three of 26 patients (88.5%) had a trunk shift more than 2.0 cm away from the midline, showing a poor coronal balance, although both the lumbosacral and the proximal curves were no more than 35°. With respect to the sagittal profile at presentation, the mean kyphotic angle of the thoracic spine was 17.4° (range, 2-47°). It was noted that the incidence of thoracic hypokyphosis was 84.6% (17/26). The mean lumbar lordotic angle was 22.1° (range, 0-49°), hence the lumbar hypolordosis was demonstrated in all the 26 patients.

**Table 2:** Preoperative and postoperative radiographic findings of patients

**Patient No**.	Age (yrs)	Gender	Level of disc herniation	Side of disc herniation	Trunk shift(cm)		Thoracic or thoracolumbar curve				Lumbosacral curve			
					
					Pre-op	Post-op	Levels	Direction	Magnitude (degrees)		Levels	Direction	Magnitude (degrees)	
												
									Pre-op	Post-op			Pre-op	Post-op
1	16.3	F	L4-L5	L	4.6	1.6	T4-L2 (11)	L	28	13	L2-S1 (5)	R	15	11
2	17.1	M	L4-L5	R	4.8	1.7	T6-L3 (10)	L	31	13	L3-S1 (4)	R	20	8
3	19.8	M	L4-S1	R	-3.0	1.5	T6-L3 (10)	L	20	8	L3-S1 (4)	R	21	10
4	19.5	F	L4-L5	R	5.4	2.0	T3-L2 (12)	L	25	7	L2-S1 (5)	R	21	9
5	17.7	M	L4-L5	R	2.2	1.1	T8-L3 (8)	L	19	9	L3-S1 (4)	R	10	9
6	16.4	F	L5-S1	L	6.6	-0.5	T3-L3 (13)	L	24	11	L3-S1 (4)	R	26	10
7	16.8	M	L4-L5	R	0.9	0.5	T3-L3 (13)	L	17	8	L3-S1 (4)	R	10	3
8	14.7	M	L5-S1	L	-3.2	-1.4	T6-L2 (9)	R	16	9	L2-S1 (5)	L	15	10
9	18.0	M	L4-L5	L	-3.8	1.7	T5-L4 (12)	R	21	9	L4-S1 (3)	L	14	6
10	20.0	M	L4-L5	L	-3.6	-0.5	T5-L2 (10)	R	25	13	L2-S1 (5)	L	20	9
11	19.3	M	L4-S1	L	-4.9	-1.1	T5-L2 (10)	R	27	13	L2-S1 (5)	L	24	8
12	16.2	F	L5-S1	L	2.1	1.1	T7-L4 (10)	R	20	11	L4-S1 (3)	L	28	5
13	14.8	F	L4-S1	L	-5.8	-2.3	T4-L2 (11)	R	30	12	L2-S1 (5)	L	20	7
14	14.3	M	L4-S1	L	2.9	1.8	T8-L2 (7)	L	25	8	L2-S1 (5)	R	16	10
15	19.1	M	L5-S1	L	-3.3	-1.2	T3-L2 (12)	R	28	9	L2-S1 (5)	L	18	11
16	18.4	M	L5-S1	R	7.7	-0.9	T2-L2 (13)	L	32	11	L2-S1 (5)	R	27	7
17	16.5	M	L4-L5	L	-2.6	0.6	T5-L4 (12)	R	15	7	L4-S1 (3)	L	12	8
18	19.0	F	L4-L5	R	-3.4	-1.7	T5-L2 (10)	R	27	10	L2-S1 (5)	L	23	6
19	19.2	M	L5-S1	L	-3.2	-0.8	T2-L3 (14)	R	26	13	L3-S1 (4)	L	26	11
20	18.4	M	L4-L5	R	3.9	1.8	T4-L2 (11)	L	23	11	L2-S1 (5)	R	21	9
21	18.7	M	L4-L5	L	-1.8	0.6	T5-L2 (10)	R	32	13	L2-S1 (5)	L	29	11
22	19.6	M	L4-S1	L	-3.6	-0.7	T5-L2 (10)	R	14	7	L2-S1 (5)	L	15	5
23	17.2	F	L4-L5	R	3.2	1.9	T7-L3 (9)	L	35	11	L3-S1 (4)	R	19	9
24	18.5	M	L4-S1	L	-4.3	-1.5	T4-L2 (11)	R	30	12	L2-S1 (5)	L	23	11
25	19.6	M	L4-L5	L	-4.5	-1	T3-L1 (11)	R	32	13	L1-S1 (6)	L	25	11
26	14.4	F	L4-L5	L	1.2	0.7	T8-L3 (8)	R	20	10	L3-S1 (4)	L	10	6

R = right; L= left; Pre-op= preoperative; Post-op= postoperative

84.6% (22/26) patients had a disc herniation at the convex side of lumbosacral curve (Table 3). There was a statistically significant association between the direction of the curve and the side of the disc herniation according to Fisher's exact test (P = 0.001). Similarly, 73.1% (19/26) patients showed a trunk shift toward the opposite side of disc herniation (Table 4), and an apparent correlation between the direction of trunk shift and the side of disc herniation was noted (P = 0.038).

**Table 3:** Association between the side of lumbosacral curve and the side of disc herniation

Side of lumbosacral curve	Side of disc herniation		
	
	Left	Right	Total
Left	14	1	15
Right	3	8	11
Total	17	9	26

Fisher's exact test, P = 0.001.

**Table 4:** Association between the direction of trunk shift and the side of disc herniation

Direction of trunk shift	Side of disc herniation		
	
	Left	Right	Total
Left	5	7	12
Right	12	2	14
Total	17	9	26

Fisher's exact test, P = 0.038.

Follow-up data were obtained from clinic follow-up visits. The final follow-up time in these patients varied between 6 months and 54 months after surgery (mean, 29.4 months). 17 of 26 patients had a follow-up more than two years (2-4.5 years). The mean improvement of pain in terms of numeric pain scale was 82.9% (range, 56-100%) immediately after operation, and 73.1% (range, 40-100%) at the last follow-up. No patients need pain medication at follow-up. The mean ODI was 21.4% (range, 11-62%) before surgery, improved to 7.3% (range, 0-36%) at the final follow-up. Immediately after surgery, the mean thoracic or thoracolumbar curve decreased from 24.7° (range, 14-35°) to 10.4° (range, 7-13°), and the mean lumbosacral curve improved from 19.5° (range, 10-29°) to 8.5° (range, 3-11°). There were significant differences between preoperative and postoperative values in terms of the Cobb angle of thoracic/thoracolumbar and lumbosacral curves (P < 0.05). The mean trunk shift decreased from 3.7 cm (range, 0.9-7.7 cm) to 1.2 cm (range, 0.5-2.3 cm). The mean thoracic kyphotic angle improved from 17.4° (range, 2-47°) to 27.6° (range, 18-41°) and the mean lumbar lordotic angle increased from 22.1° (range, 0°-49°) to 32.2° (range, 15-59°) at the immediate postoperative period. Similarly, there were significant differences between preoperative and postoperative values in terms of the thoracic kyphotic angle and lumbar lordotic angle (P < 0.05). In the 17 patients with a follow-up more than two years, none had the residual proximal curve greater than 20°, while a residual lumbosacral curve ≥20° was only noticed in two patients (No. 12 and 21). Those two patients had a relatively large Cobb angle of the lumbosacral curve before surgery. No associations between pain, residual curve angle and sagittal profile were noticed since both of them were pain-free and the improvement of lumbar lordosis was well maintained during follow-up. At the final follow-up, the AVR in the residual lumbosacral curve was Grade 0 in patient No. 12 and Grade 1 in patient No. 21, respectively. And the lumbosacral curves in the two patients resolved during the Adams forward bend test, showing a nonstructural pattern. As for the three patients with Risser grade of 3 (No. 8, 14 and 26), none of them had the residual curve more than 20° at the time of skeletal maturity. The Cobb angle of proximal curve was averaged at 11° (range, 5-16°), while the mean magnitude of lumbosacral curve increased slightly to 10.5° (range, 3-22°). However, no significant difference was demonstrated as compared with the postoperative values (P > 0.05). And the coronal balance was well maintained with an average trunk shift as 0.9 cm (range, 0.5-1.7 cm). Regarding the sagittal profile, the mean thoracic kyphotic angle was 25.8° (range, 19-35°) and the mean lumbar lordotic angle was found to be 37.4° (range, 27-56°). Also, no significant difference was revealed as compared with the postoperative values (P > 0.05).

## Discussion

Scoliosis is a musculoskeletal disorder in which there is a lateral curvature of the spine. In most young individuals, the scoliosis is idiopathic, meaning that it is not known what has caused the curve to occur. However, it may also occur as a part or complication of an underlying health condition, such as neuromuscular disorders, lower limb discrepancy, as well as LDH. Although scoliotic posture has been reported to be a frequent symptom in adolescents with LDH [3,17-19], most patients of lumbar disc disease in this age group may be underdiagnosed at the initial visit for evaluation of scoliotic posture, which could be attributed to the rare prevalence and the more nonspecific features of LDH in adolescents as compared with those seen in adults. In our series, there were 4 patients (No. 6, 13, 14, and 24) having been misdiagnosed as adolescent idiopathic scoliosis (AIS) treated with bracing. Hence, careful clinical assessment is crucial to avoid misdiagnosis and prevent undesirable results from inappropriate management. In the current study, all the patients initially presented scoliotic posture for clinical evaluation, while the other symptoms were not well expressed by the patients attributable to their young age or the nonspecific features of the symptoms. However, following thorough history-taking and careful physical examination, some clinical findings related to LDH were revealed (Table 1). Furthermore, we found that limitations of daily activities were more predominant than the focal findings such as sensory deficit or motor weakness, and the leg pain often ran down to the thigh rather than to the foot. An Adams forward bend test can usually distinguish between postural and structural scoliosis. In the present study, this test was performed on 7 patients having no restriction of forward flexion at presentation. All of them showed a disappearing of the curve during the test, demonstrating a nonstructural pattern.

To our knowledge, the curve patterns and clinical courses of scoliotic posture secondary to LDH have not been well documented in adolescents. In the current study, some unique radiographic characteristics of the scoliotic curve were noticed, which were typically different from those of AIS. All these 26 patients showed a typical curve pattern featured by a short and fractional lumbosacral curve accompanied with a long thoracic or thoracolumbar curve toward the opposite side. The lumbosacral curve involved 4.5 levels on average, while an average 10.7 levels were entangled in the proximal curve. Most of the proximal curves (80.7%) were so irregular that the apical vertebrae could hardly be identified properly (Figure 1). The vertebral wedging in the apical area was not demonstrated, and all the vertebrae in the apical area had slight rotation (no more than Grade 1) or neutral position. As for the double curve pattern in AIS, right thoracic and left lumbar components are common. Both the thoracic and lumbar curves are regular and the apical vertebral body wedging can be commonly observed. The end vertebrae and apexes for this type of AIS are typical as following [20]: thoracic component being from T4-T6 to T11 with an apex at T8 or T9, and lumbar component from T11 to L3 or L4 with an apex at L2. Secondly, our study showed that a majority of patients had a small curve size while assosiated with a significant coronal imbalance. The mean preoperative Cobb angle of lumbosacral curve was 19.5° (range, 10°-29°), and the curve magnitude of proximal thoracic or thoracolumbar curve was averaged at 24.7° (range, 14°-35°). However, a mean trunk shift of 3.7 cm was noted, and 23 patients (88.5%) showed a poor coronal balance with the trunk shift more than 2.0 cm away from the midline. On the contrary, the coronal imbalance may seldom occur in AIS with double curve pattern as the Cobb angle is less than 35°. Thirdly, a relatively straight sagittal profile was noted. All of the patients had a lumbar hypolordosis, and 84.6% patients showed a thoracic hypokyphosis. Finally, there was a statistically significant association between the direction of the scoliosis and the side of the disc herniation (P = 0.001). Nineteen patients (73.1%) showed a trunk shift toward the opposite side of disc herniation (P = 0.038). In addition, curve flexibility on the basis of lateral bending films is one of the important radiographic characteristics of the scoliosis and is crucial to distinguish between structural and posture curves, which we propose should be done in those patients without severe back pain. We acknowledge the lack of assessment of the curve flexibility for those patients complaining of low back pain or buttock pain in the current study.

The mechanical relationship between scoliotic posture and LDH is still not very clear. Finneson [21] speculated that the scoliotic posture would go toward the opposite side of the sciatica to decrease the nerve root stimulation, if the herniation is lateral to the nerve root. In contrast, the lumbar list is toward the side of the sciatica to diminish nerve compression, when the herniation is located medial to the nerve root. However, some subsequent studies have reported that their clinical results contradicting the Finneson's hypothesis [1,2,22]. Suk et al [1] reported their findings in 45 patients with a mean age of 31.2 years (13-62 years), showing the direction of scoliotic posture was not associated with the location of nerve root compression, while 66.7% (30/45) patients had a disc herniation at the convex side of scoliotic posture, demonstrating a significant association between the direction of scoliotic posture and the side of disc herniation. Therefore, Suk et al [1] attributed this phenomenon to an autonomic decompression mechanism that the herniated disc was thought to be reduced in size by stretching or inward bulging at the convex side of the scoliotic posture. In the current study, disc herniation occurred unilaterally in all the patients. With regard to the 6 patients with L4-S1 herniation, the two-level herniated discs located on the same side were noticed in each patient, which might be attributed to the fact that the patients included were a special group who presented scoliotic posture as their initial symptom. 84.6% (22/26) patients had a disc herniation at the convex side of lumbosacral curve, showing a higher incidence than that seen in a wide age-ranged population by Suk et al [1]. Based on adolescent individuals recruited exclusively in the current study, we believe the higher incidence of disc herniation at the convex side of lumbosacral curve in young patients should be resulted from the anatomical feature that pediatric spines have better adaptive capacity to protect nerve tissue via lateral flexion. In addition, we investigated the coronal balance in relation to disc herniation in these patients, which was not reported in detail by previous studies. We found that a statistically significant correlation between the direction of trunk shift and the side of disc herniation (P = 0.038). We propose that a trunk shift toward the opposite side of disc herniation can change the weight-bearing on legs, a small amount of weight supported by the affected leg may alleviate the nerve root irritation.

Published studies have reiterated that adolescents, as opposed to adults, are less responsive to conservative treatment of LDH [4,10,17], which is mainly attributed to the high elasticity and viscosity of the disc material in adolescents compared with that in adults [23]. Kurihara et al reviewed 70 adolescents with lumbar disc herniation [18]. Only 40% of the patients responded to conservative treatment and recurrence of symptoms was common. The goal of the management of LDH in adolescents is to relieve symptoms and allow early return to school education and social interaction. This has not only physical consequences but a psychological effect on adolescent patients. Hence, conservative treatment should be brief for patients with persisting disability and even for those having no predominant neurologic signs [18]. Surgical intervention could be considered earlier for achieving a quicker recovery with fewer complications [24]. Borgesen and Vang reviewed 158 pediatric patients, and noted good to excellent results after surgery in 93.7% of the patients [25]. In our series, 21 patients didn't response well to conservative management for 7-12 weeks, a sequential posterior discectomy was carried out. Although the other 5 patients achieved pain relief after conservative treatment, the persistence of their scoliotic posture and trunk shift was still noted. A decision to operate was made considering that the scoliotic posture and trunk shift had not only physical consequences but a negative psychological effect on adolescent patients. In addition, the likelihood that the persistent curve might progress into a structural scoliosis was also taken into consideration for surgical decision-making. After surgery, the immediate mean improvement of pain was noted as 82.9%, and no patients needed pain medication at follow-up.

Matsui et al [2] performed posterior discectomy on 40 children and adults with lumbar disc herniation and scoliotic posture. The mean Cobb angle decreased from 10.7° (3°-34°) before surgery to 2.7° within an average of 7.5 months after surgery. Similarly, in a prospective study by Suk et al [1], the immediate improvement of the mean Cobb angle from 9.8° (5°-25°) to 1.8° (0°-14°) following conventional open discectomy was noted in 45 patients suffering from disc herniation and scoliosis. However, both the aforementioned studies [1,2] involved a wide age-ranged population including children and adults, and some cases had a Cobb angle less than 10°. As for the present study, the preoperative curve magnitude was relatively larger. Considering the more severe preoperative deformity and coronal imbalance in our series, some additional postsurgical conservative measures, such as posture training with a corset, were performed to maximize the outcome. All of the patients had an immediate curve improvement. The mean Cobb angle of lumbosacral curve was 19.5° at presentation, decreased to 8.5° immediately after surgery. Accordingly, the proximal curve diminished from 24.7° to 10.4°. Curve stabilization was noticed in the 17 patients with a more than 2-year follow-up. The Cobb angle of the proximal curve was averaged at 11° and the mean magnitude of lumbosacral curve was 10.5°. The coronal balance was well maintained with a trunk shift as 0.9 cm. At the last follow-up, only two patients still had a residual lumbosacral curve greater than 20°. We conclude that earlier discectomy and adjunct postsurgical conservative measures can provide a greater opportunity for correction and stabilization of scoliotic posture. In the present study, a discectomy was considered in those patients failed to respond to conservative measures for 7-12 weeks. For those patients who have a residual curve, especially still with some growth potential, the curve should be closely observed till skeletal maturity.

## Conclusion

Some unique radiographic characteristics of the scoliotic posture were noticed in our series, which were typically different from those of AIS. We also found the direction of lumbosacral curve and trunk shift was related to the side of disc herniation. Earlier discectomy followed by adjunct conservative measures would provide satisfactory outcomes. Spontaneous correction of scoliotic posture could be achieved immediately after surgery and well maintained during follow-up. However, we should emphasize that the findings above were revealed only in a group of adolescents having surgery after failed conservative management, while those who responded well to conservative treatment were not included in the current study.

## Competing interests

The authors declare that they have no competing interests.

## Authors' contributions

ZZ carried out this study and drafted the manuscript. QZ performed the statistical analysis. BW, YY, BQ and YD helped to draft the manuscript. QY conceived of the study, participated in its design and coordination. All authors read and approved the final manuscript.

## Pre-publication history

The pre-publication history for this paper can be accessed here:

http://www.biomedcentral.com/1471-2474/12/216/prepub

## References

[B1] Suk KS Lee HM Moon SH Kim NH Lumbosacral scoliotic list by lumbar disc herniation Spine 2001 26 667 671 10.1097/00007632-200103150-00023 11246383

[B2] Matsui H Ohmori K Kanamori M Ishihara H Tsuji H Significance of sciatic scoliotic list in operated patients with lumbar disc herniation Spine 1998 23 338 342 10.1097/00007632-199802010-00010 9507622

[B3] Pinto FC Poetscher AW Quinhones FR Pena M Taricco MA Lumbar disc herniation associated with scoliosis in a 15-year-old girl: case report Arq Neuropsiquiatr 2002 60 295 298 10.1590/S0004-282X2002000200022 12068364

[B4] Kumar R Kumar V Das NK Behari S Mahapatra AK Adolescent lumbar disc disease: findings and outcome Childs Nerv Syst 2007 23 1295 1299 10.1007/s00381-007-0370-1 17541606

[B5] Bradbury N Wilson LF Mulholland RC Adolescent disc protrusions. A long-term follow-up of surgery compared to chymopapain Spine 1996 21 372 377 10.1097/00007632-199602010-00024 8742215

[B6] Adams MA Roughley PJ What is intervertebral disc degeneration, and what causes it? Spine 2006 31 2151 2161 10.1097/01.brs.0000231761.73859.2c 16915105

[B7] Battie MC Videman T Lumbar disc degeneration: epidemiology and genetics J Bone Joint Surg Am 2006 88 Suppl 2 3 9 16595435 10.2106/JBJS.E.01313

[B8] Martinez-Lage JF Fernandez Cornejo V Lopez F Poza M Lumbar disc herniation in early childhood: case report and literature review Childs Nerv Syst 2003 19 258 260 12715195 10.1007/s00381-003-0720-6

[B9] Mattila VM Saarni L Parkkari J Koivusilta L Rimpela A Early risk factors for lumbar discectomy: an 11-year follow-up of 57,408 adolescents Eur Spine J 2008 17 1317 1323 10.1007/s00586-008-0738-2 18682991 PMC2556481

[B10] Fakouri B Nnadi C Boszczyk B Kunsky A Cacciola F When is the appropriate time for surgical intervention of the herniated lumbar disc in the adolescent? J Clin Neurosci 2009 16 1153 1156 10.1016/j.jocn.2009.01.023 19546005

[B11] Bezer M Erol B Kocaoglu B Aydin N Guven O Low back pain among children and adolescents. Acta Orthop Traumatol Turc 2004 38 136 144 15129033

[B12] Edwards CC Lenke LG Peelle M Sides B Rinella A Bridwell KH Selective thoracic fusion for adolescent idiopathic scoliosis with C modifier lumbar curves: 2- to 16-year radiographic and clinical results Spine 2004 29 536 546 10.1097/01.BRS.0000109992.22248.77 15129068

[B13] Puno RM An KC Puno RL Jacob A Chung SS Treatment recommendations for idiopathic scoliosis: an assessment of the Lenke classification Spine 2003 28 2102 2114 discussion 2114-2105. 10.1097/01.BRS.0000088480.08179.35 14501921

[B14] Qiu Y Zhu Z Wang B Yu Y Qian B Zhu F Radiological presentations in relation to curve severity in scoliosis associated with syringomyelia J Pediatr Orthop 2008 28 128 133 10.1097/bpo.0b013e31815ff371 18157058

[B15] Spiegel DA Flynn JM Stasikelis PJ Dormans JP Drummond DS Gabriel KR Loder RT Scoliotic curve patterns in patients with Chiari I malformation and/or syringomyelia Spine 2003 28 2139 2146 10.1097/01.BRS.0000084642.35146.EC 14501926

[B16] Zheng GXZX Liu GL Reliability of the Modified Oswestry Disability Index for evaluating patients with low back pain Chinese Journal of Spine and Spinal Cord 2002 12 3

[B17] Ozgen S Konya D Toktas OZ Dagcinar A Ozek MM Lumbar disc herniation in adolescence Pediatr Neurosurg 2007 43 77 81 10.1159/000098377 17337916

[B18] Kurihara A Kataoka O Lumbar disc herniation in children and adolescents. A review of 70 operated cases and their minimum 5-year follow-up studies Spine 1980 5 443 451 10.1097/00007632-198009000-00009 7455775

[B19] Lee DY Ahn Y Lee SH Percutaneous endoscopic lumbar discectomy for adolescent lumbar disc herniation: surgical outcomes in 46 consecutive patients Mt Sinai J Med 2006 73 864 870 17117312

[B20] Coonrad RW Murrell GA Motley G Lytle E Hey LA A logical coronal pattern classification of 2,000 consecutive idiopathic scoliosis cases based on the scoliosis research society-defined apical vertebra Spine 1998 23 1380 1391 10.1097/00007632-199806150-00016 9654630

[B21] Finneson BE Low Back Pain PhiladelphiaJB Lippincott 1973 290 303

[B22] Lorio MP Bernstein AJ Simmons EH Sciatic spinal deformity-lumbosacral list: an "unusual" presentation with review of the literature J Spinal Disord 1995 8 201 205 10.1097/00002517-199506000-00004 7670210

[B23] Gennuso R Humphreys RP Hoffman HJ Hendrick EB Drake JM Lumbar intervertebral disc disease in the pediatric population Pediatr Neurosurg 1992 18 282 286 10.1159/000120676 1476937

[B24] Ishihara H Matsui H Hirano N Tsuji H Lumbar intervertebral disc herniation in children less than 16 years of age. Long-term follow-up study of surgically managed cases Spine 1997 22 2044 2049 10.1097/00007632-199709010-00022 9306537

[B25] Borgesen SE Vang PS Herniation of the lumbar intervertebral disk in children and adolescents Acta Orthop Scand 1974 45 540 549 10.3109/17453677408989177 4281255

